# JAK3 Is Expressed in the Nucleus of Malignant T Cells in Cutaneous T Cell Lymphoma (CTCL)

**DOI:** 10.3390/cancers13020280

**Published:** 2021-01-14

**Authors:** Chella Krishna Vadivel, Maria Gluud, Sara Torres-Rusillo, Lasse Boding, Andreas Willerslev-Olsen, Terkild B. Buus, Tea Kirkegaard Nielsen, Jenny L. Persson, Charlotte M. Bonefeld, Carsten Geisler, Thorbjorn Krejsgaard, Anja T. Fuglsang, Niels Odum, Anders Woetmann

**Affiliations:** 1 LEO Foundation Skin Immunology Research Center, Department of Immunology and Microbiology, University of Copenhagen, 2200 Copenhagen, Denmark; cvadivel@sund.ku.dk (C.K.V.); mgluud@sund.ku.dk (M.G.); storres@sund.ku.dk (S.T.-R.); labo@ssi.dk (L.B.); awo@sund.ku.dk (A.W.-O.); terkild.buus@sund.ku.dk (T.B.B.); tea@sund.ku.dk (T.K.N.); cmenne@sund.ku.dk (C.M.B.); cge@sund.ku.dk (C.G.); thorkr@sund.ku.dk (T.K.); 2 The Danish National Biobank, Statens Serum Institut, 2300 Copenhagen, Denmark; 3 Division of Basal Tumor Biology, Department of Molecular Biology, Umea University, 90187 Umea, Sweden; jenny.persson@umu.se; 4 Department of Biomedical Sciences, Malmo University, 21428 Malmo, Sweden; 5 Department of Plant and Environmental Sciences, University of Copenhagen, 1871 Frederiksberg, Denmark; atf@plen.ku.dk

**Keywords:** JAK3, cutaneous T cell lymphoma, Mycosis fungoides, Sézary syndrome, tyrosine kinases

## Abstract

**Simple Summary:**

JAK3 plays an important role in the pathogenesis of cutaneous T cell lymphoma. JAK3 belongs to the Janus kinase family of receptor-associated tyrosine kinases located in cytoplasm adjacent to the plasma membrane. In this study, we show that JAK3 can also be ectopically expressed in the nucleus in CTCL cell lines and primary cells from CTCL patients. Importantly, JAK3 interacts with the nuclear protein RNA polymerase II and phosphorylates Histone H3. Thus, our data provide first evidence for nuclear expression of JAK3 and interactions with key nuclear proteins in malignant T cells suggesting a novel non-canonical role in CTCL.

**Abstract:**

Perturbation in JAK-STAT signaling has been reported in the pathogenesis of cutaneous T cell lymphoma (CTCL). JAK3 is predominantly associated with the intra-cytoplasmic part of IL-2Rγc located in the plasma membrane of hematopoietic cells. Here we demonstrate that JAK3 is also ectopically expressed in the nucleus of malignant T cells. We detected nuclear JAK3 in various CTCL cell lines and primary malignant T cells from patients with Sézary syndrome, a leukemic variant of CTCL. Nuclear localization of JAK3 was independent of its kinase activity whereas STAT3 had a modest effect on nuclear JAK3 expression. Moreover, JAK3 nuclear localization was only weakly affected by blockage of nuclear export. An inhibitor of the nuclear export protein CRM1, Leptomycin B, induced an increased expression of SOCS3 in the nucleus, but only a weak increase in nuclear JAK3. Importantly, immunoprecipitation experiments indicated that JAK3 interacts with the nuclear protein POLR2A, the catalytic subunit of RNA Polymerase II. Kinase assays showed tyrosine phosphorylation of recombinant human Histone H3 by JAK3 in vitro—an effect which was blocked by the JAK inhibitor (Tofacitinib citrate). In conclusion, we provide the first evidence of nuclear localization of JAK3 in malignant T cells. Our findings suggest that JAK3 may have a cytokine-receptor independent function in the nucleus of malignant T cells, and thus a novel non-canonical role in CTCL.

## 1. Introduction

Cutaneous T cell lymphoma (CTCL) is a haematological cancer where clonally expanded malignant T cells accumulate in the skin leading to chronically inflamed skin lesions [1,2,3,4,5]. The disease presents with skin patches, plaques and lesions and becomes progressive disease in approximately a third of the patients [5,6]. Mycosis fungoides (MF) and Sézary syndrome (SS) are the two well-studied clinically distinct variants of CTCL [2,5]. MF is the most common form that mainly affects the skin and often has a protracted and relatively indolent disease course, whereas SS is a rare, but highly aggressive, leukemic variant of CTCL with generalized skin involvement [3]. Despite comprehensive research, the aetiology of CTCL remains largely unknown. Single-cell sequencing has revealed a high level of disease heterogeneity—both between patients (inter-patient heterogeneity) and in the individual patient (intra-patient heterogeneity) [7] indicating that individual patients harbour highly heterogeneous populations of malignant T cells [7,8] which has also been confirmed by immunohistochemistry and analyses of separate lesions derived from the same patient [9,10]. Other genetic studies have also indicated that CTCL is a highly heterogeneous disease and is not driven by a single somatic mutation [11,12].

Deregulation of common signaling pathways is a characteristic feature of malignant T cells and implicated in disease progression [13]. In particular, deregulated Janus Kinase 3 (JAK3) signaling and activation of downstream targets such as STAT3, STAT5, and STAT6 appears to play an important role in cytokine expression, proliferation, and resistance to apoptosis in malignant T cells [14,15,16,17]. Aberrant JAK3/STAT activation indirectly impact multiple other signaling pathways through modulators such as microRNAs (miRs) [18]. JAK3/STAT signaling drives the expression of oncogenic miRs such as miR-155, miR-21, miR-93, and miR-214 [19,20] as well as repression of putative tumour suppressors (SATB1, PDCD4, JARID2) in malignant T cells [18,21,22]. Interestingly, activating mutations in JAK3 and its substrates STAT3 and STAT5 as well as deletions and/or loss of function of negative regulators such as SOCS1, SOCS3, and HNRNPK have been reported in CTCL [12,23,24,25]. Moreover, environmental factors such as *Staphylococcus aureus* can fuel disease activity through an enhanced JAK/STAT activation, cytokine receptor expression, and proliferation of malignant T cells in situ in patients with severe CTCL. This suggest that a series of different events and factors may converge to trigger deregulated JAK/STAT signaling in malignant T cells highlighting the key role of JAK3 and downstream effectors in carcinogenesis in CTCL and other T cell malignancies [26,27,28]. Conventionally, Janus kinases are considered to be members of the class of receptor-associated tyrosine kinases (reviewed in [29]). Surprisingly, recent studies reported on nuclear expression of JAK1 in large B cell lymphoma and JAK2 was shown to function as nuclear tyrosine kinase regulating histone phosphorylation and cellular survival in human haematopoietic stem cells and B cell leukemia cells [30,31]. Yet, nothing is known about nuclear functions of JAK3 and to our knowledge no studies so far have reported on nuclear expression and function of Janus kinases in malignant T cells. Accordingly, we investigated the nuclear expression of JAK3 and whether it interacts with nuclear proteins in malignant T cells.

## 2. Results

As mentioned above, JAK3 is believed to play an important role in the pathogenesis of CTCL. Thus, JAK3 promotes survival and proliferation of malignant T cells and expression of proto-oncogenes/oncomiRs and cytokines (IL-5, IL-9, IL-13, IL-17F and LTA), some of which are growth factor to malignant T cells while others modulate the tumour micro-environment (TME) and anti-cancer immunity (Summarized in Table S1). To confirm the relevance of JAK3 inhibition in the present cellular context, MyLa2059 cells were treated with JAK3 inhibitor or vehicle control and the fold change in mRNA expression in vehicle-to-JAK3 inhibitor-treated cells is shown confirming that JAK3 regulates expression of these genes in MyLa2059 cells (Table S1).

**Figure 1:**
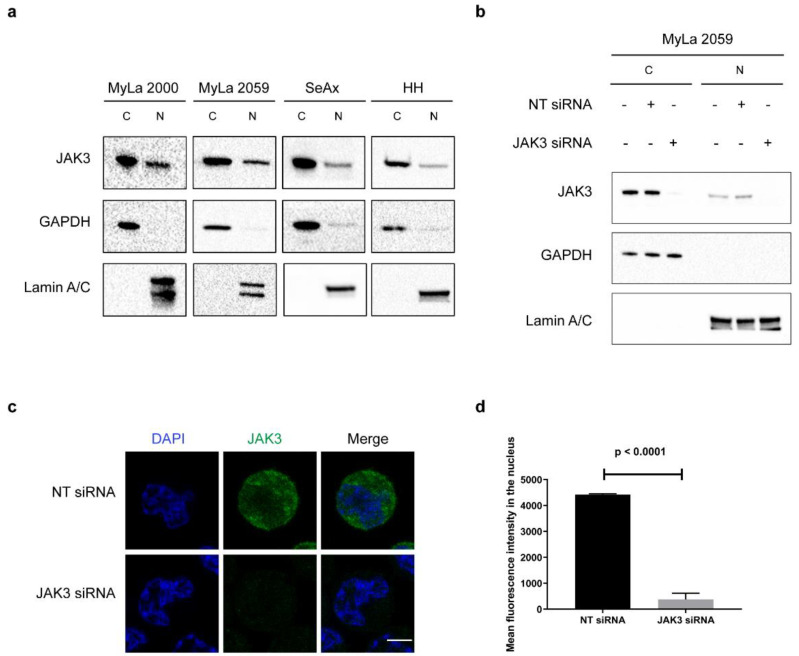
JAK3 is expressed in the nucleus in CTCL cell lines. (**a**) Immunoblot shows JAK3 expression in the cytoplasmic and nuclear extracts of malignant cell lines MyLa 2000, MyLa 2059, SeAx, and HH. (**b**) Immunoblot shows low or no expression of JAK3 in the cytoplasmic (C) and nuclear extracts (N) of MyLa 2059 after 48 h of siRNA mediated knockdown of JAK3. (**c**) Immunofluorescence (IF) of MyLa 2059 confirming JAK3 expression in the nucleus. Scale bar = 5 μm. NT siRNA is the non-targeted control siRNA and JAK3 siRNA is the siRNA targeted against *JAK3*. (**d**) Mean fluorescence intensity of JAK3 calculated in the nucleus of MyLa 2059 after IF. Error bar represents standard error of mean.

Nuclear localization of JAK3 has recently been observed in HIV-infected CD4^+^ T cells [32] which prompted us to hypothesize that malignant CD4^+^ T cells from CTCL patients may also display ectopic expression and function of JAK3. Accordingly, we performed Western blotting (WB) on isolated cytoplasmic and nuclear extracts from the malignant cell lines MyLa 2000, MyLa 2059, SeAx, and HH. As shown in Figure 1a, JAK3 was expressed in the nucleus of all of four tested malignant cell lines (Figure 1a). We next performed transient knockdown of JAK3 using siRNA against *JAK3* to validate the specificity of the anti-JAK3 antibody prior to analysis for nuclear JAK3 expression by immunofluorescence. As shown in Figure 1b and Figure S1, siRNA treatment for 48 h almost completely abolished nuclear JAK3 expression as judged from WB on cytoplasmic and nuclear extracts from the MyLa2059 and MyLa 2000 respectively. In parallel, we performed immunofluorescence in MyLa2059. JAK3 expression was almost completely abolished by siRNA-mediated JAK3 knock-down (Figure 1c) when compared with cells treated with the scrambled siRNA control (Figure 1c). JAK3 was expressed in the nucleus as judged from the merged images of nuclear and JAK3 staining (Figure 1c), and almost completely absent following siRNA mediated JAK3 knockdown (Figure 1c). Quantification of mean fluorescence intensity (MFI) confirmed the nuclear expression of JAK3 in MyLa 2059, which was profoundly inhibited following siRNA mediated JAK3 knockdown (Figure 1d, *p* < 0.0001). We also performed immunofluorescence staining in MyLa 2000 and verified the nuclear expression of JAK3 (Figure S1).

**Figure 2:**
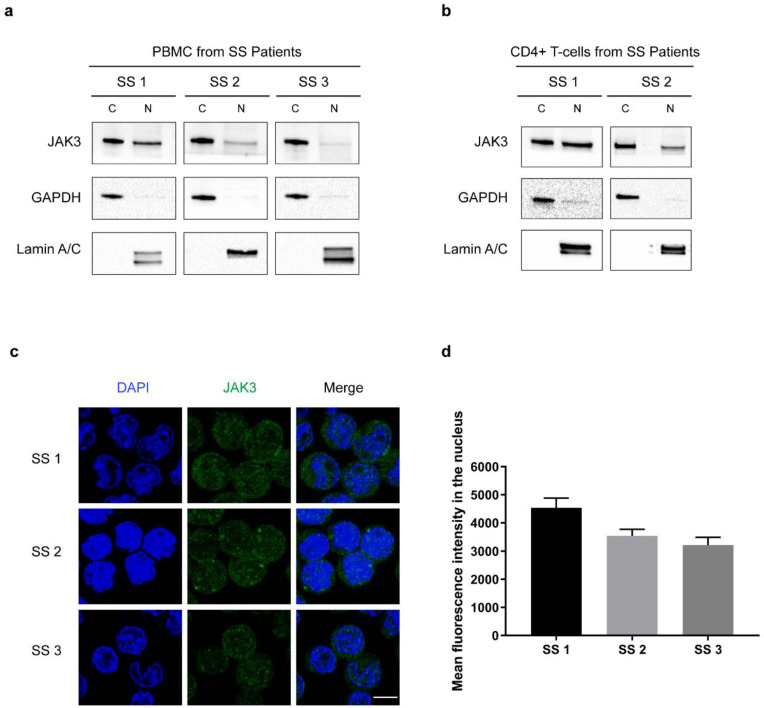
JAK3 is expressed in the nucleus in cells from SS patients. (**a**) Immunoblot shows JAK3 expression in the cytoplasmic and nuclear extracts of PBMC isolated from SS patients. (**b**) Immunoblot shows JAK3 expression in the cytoplasmic (C) and nuclear extracts (N) of CD4^+^ T cells isolated from SS patients. (**c**) Immunofluorescence (IF) confirming JAK3 expression in the nucleus of CD4^+^ T cells isolated from SS patients. Scale bar = 5 μm. (**d**) Mean fluorescence intensity of JAK3 calculated in the nucleus after IF. Error bar represents standard error of mean. Display settings were adjusted after image analysis using Image J and the same settings were propagated in all samples.

To address whether nuclear JAK3 expression was a common feature of malignant T cells, we extracted cytoplasmic and nuclear lysates and performed WB using PBMC from patients with SS. Immunoblots showed that JAK3 was expressed in the nuclear extracts (Figure 2a) extending our findings above in the malignant T cell lines. We also performed nuclear and cytoplasmic extraction in PBMC from healthy individuals showing low expression of JAK3 in the nucleus (Figure S2) confirming other studies by Ivan L et al. that healthy T cells may also display nuclear JAK3 expression [32]. Next, we separated CD4^+^ T cells from PBMC isolated from SS patients and extracted cytoplasmic and nuclear extracts. Immunoblots of lysates show that JAK3 was expressed in the nucleus of CD4^+^ T cells isolated from SS patients (Figure 2b). These findings were further substantiated by JAK3 immunofluorescence analysis in primary malignant T cells from three SS patients (Figure 2c,d).

**Figure 3:**
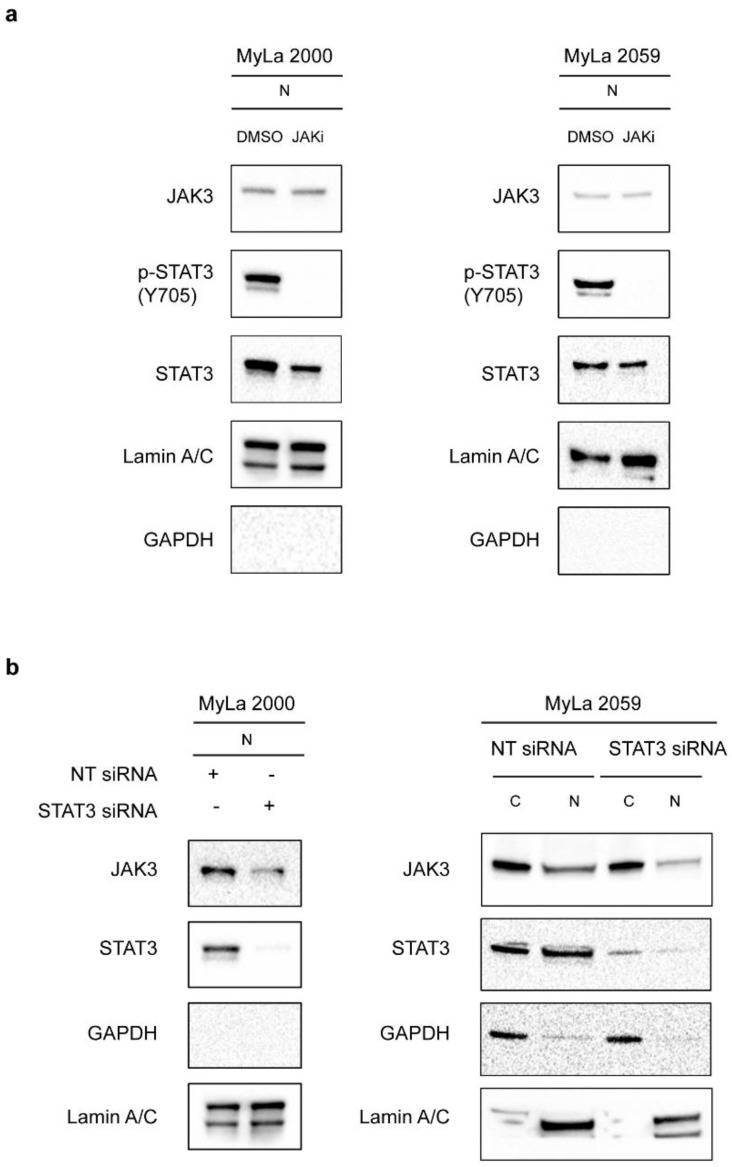
Nuclear localization of JAK3 is independent of its kinase activity and only partly dependent on STAT3. (**a**) Immunoblot shows JAK3 expression in the nuclear extracts (N) of MyLa 2000 (left) and MyLa 2059 (right) after 24 h of treatment with 50 μM JAK inhibitor, Tofacitinib citrate (CP-690550-10). DMSO is used as a control. JAKi–JAK inhibitor, Tofacitinib citrate (CP-690550-10). (**b**) Immunoblots of cytoplasmic (C) and/or nuclear (N) lysates shows JAK3 expression in control (NT siRNA) and STAT3 transiently knocked down cells (STAT3 siRNA) in MyLa 2000 (left) and MyLa 2059 (right) after 48 h.

Once we had confirmed nuclear expression of JAK3 in CTCL, we asked whether JAK3 nuclear expression was affected by JAK3 kinase activity. Accordingly, we inhibited JAK3 activity in malignant T cell lines with the JAK inhibitor Tofacitinib citrate [33]. As an in vivo read-out of JAK3 kinase activity, we measured STAT3 tyrosine phosphorylation, a key down-stream effect of JAK in malignant T cells [34]. The expression level of JAK3 in the nucleus remained largely unchanged despite inhibition of JAK3 kinase activity, as demonstrated by a profound inhibition of phosphorylated STAT3 (Figure 3a). These findings suggests that JAK3 kinase activity is not essential for its nuclear expression. Next, we tested the hypothesis that nuclear expression of JAK3 may be influenced by STAT3. To this end, we transiently knocked down STAT3 in MyLa 2000 and MyLa 2059 using siRNA against *STAT3*, and after 48 h, we isolated cytoplasmic and nuclear extracts prior to WB. STAT3 knockdown strongly inhibited STAT3 expression in both the cytoplasmic and the nuclear fraction whereas the expression of the cytoplasmic house-keeping protein GAPDH and the nuclear protein Lamin A/C was largely unaffected (Figure 3b). STAT3 knockdown induced a modest inhibition of nuclear JAK3 expression (Figure 3b) suggesting that JAK3 expression in the nucleus was partly dependent on STAT3.

**Figure 4:**
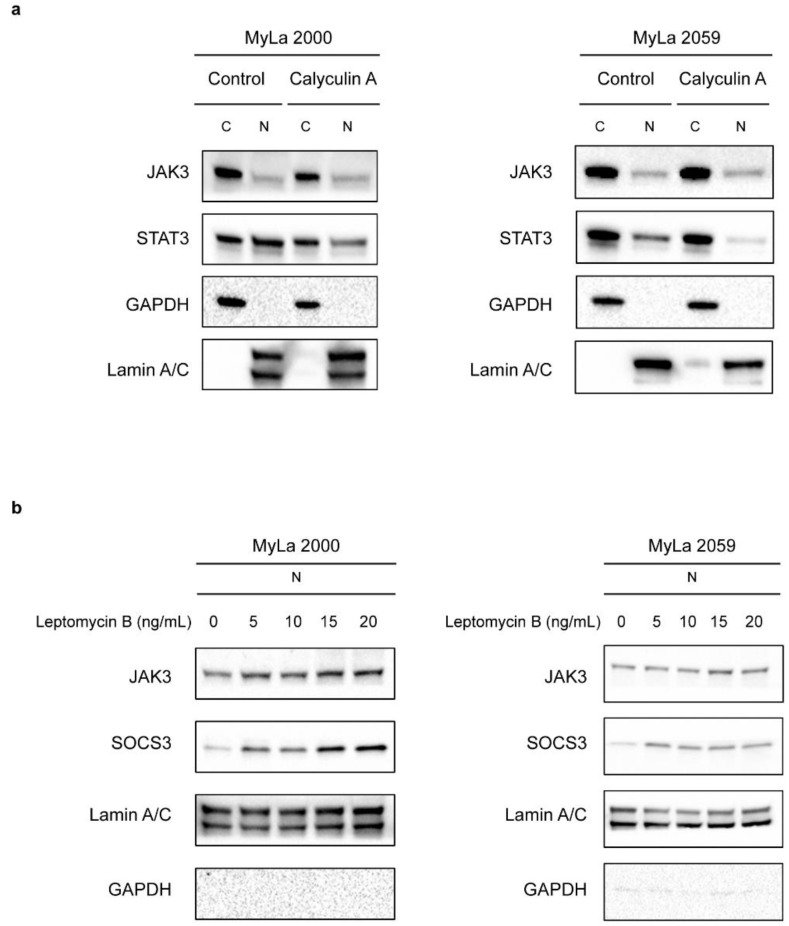
CRM1 inhibitor but not PP2A inhibitor has an effect on nuclear translocation of JAK3. (**a**) Immunoblot shows JAK3 expression in the cytoplasmic (C) and nuclear (N) extracts of MyLa 2000 (left) and MyLa 2059 (right) after treatment with either DMSO (Control) or 80 nM Calyculin A for 1 h. (**b**) Immunoblot shows JAK3 expression in the nuclear extracts (N) from MyLa 2000 (left) and MyLa 2059 (right) after treatment with mentioned concentrations (0–20 ng/mL) of Leptomycin B for 3 h.

Nuclear expression of STAT3 in malignant T cells is regulated by the ratio of its nuclear import to export, which in turn is regulated by serine/threonine phosphorylation [35]. The PP2A phosphatase inhibitor Calyculin A triggers enhanced nuclear export of STAT3 resulting in a marked decrease in nuclear STAT3 expression in malignant T cells [35]. We addressed whether JAK3 was similarly regulated. As expected, Calyculin A inhibited nuclear STAT3 expression but had no inhibitory effect on nuclear expression of JAK3 (Figure 4a) suggesting that nuclear export and expression of JAK3 and STAT3 are regulated differently.

Nuclear translocation is often mediated through a nuclear localization sequence (NLS), which has been identified in JAK1 and is required for nuclear translocation of JAK1 in hematopoietic cancer cells [36]. We looked for potential NLS sequences in JAK3 but were unable to identify a relevant sequence through NLS mapper [37]. We also analyzed primary amino acid sequence of JAK3 from UniProt [38] for potential nuclear export sequence (NES) [39] and found that the amino acid sequence of JAK3 from 163 to 170 resembles the canonical class 2 leucine-rich NES LXLXXLXL [40]. A bioinformatics NES prediction tool, NetNES [41] also predicted this site in JAK3 as a potential NES. Similarly, the NetNES also predicted a NES sequence corresponding to primary amino acid sequence from 16 to 22 (LDTSLRL) in SOCS3, a regulator of JAK-STAT signaling (reviewed in [42]). The nuclear protein CRM1/Exportin-1 recognises these NES sequences and export the cargo from the nucleus to the cytoplasm (reviewed in [43]). So, we blocked nuclear export protein CRM1 with the inhibitor, Leptomycin B [44], and investigated whether JAK3 accumulated in the nucleus after inhibition of nuclear export. Leptomycin B triggered a weak increase in JAK3 nuclear expression in MyLa 2059 and a stronger increase in MyLa 2000 cells (Figure 4b). Leptomycin B induced an increase in nuclear expression of SOCS3 in both MyLa 2000 and MyLa 2059 (Figure 4b) indicating that SOCS3 also recruits CRM1 for its nuclear export.

**Figure 5:**
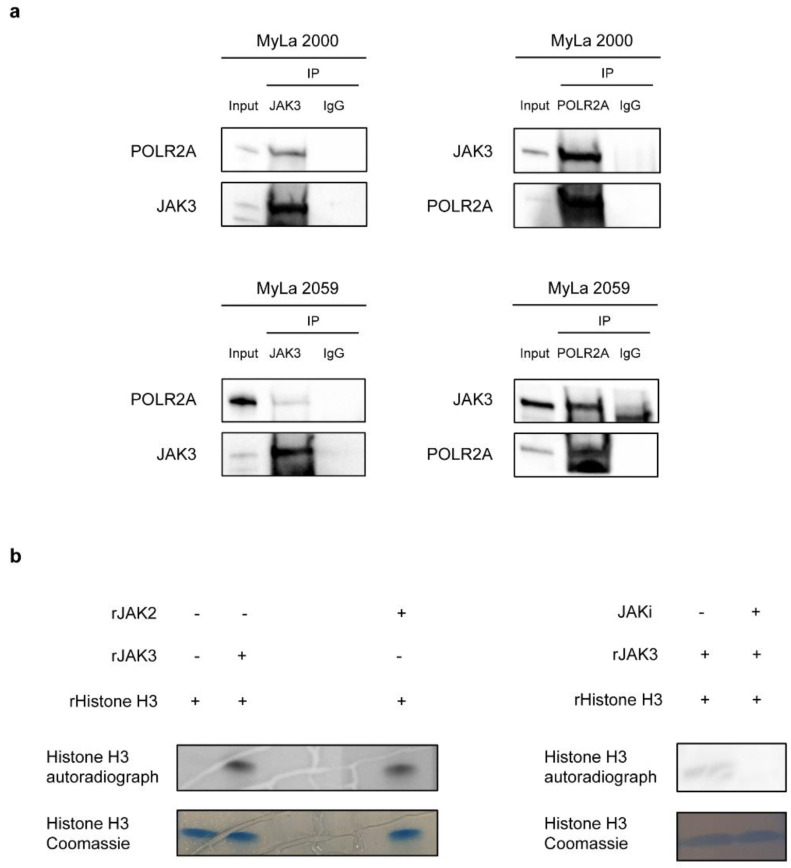
JAK3 interacts with RNA Polymerase II in MyLa cells. (**a**) Immunoblots after co-immunoprecipitation assay shows JAK3 interacts with POLR2A in MyLa 2000 (up) and MyLa 2059 (down). (**b**) Autoradiograph shows in vitro tyrosine phosphorylation of recombinant human Histone H3 by recombinant human JAK3 (left) and the phosphorylation is inhibited by pre-incubating the kinase, JAK3, with 100 nM JAK inhibitor Tofacitinib citrate (JAKi) for 30 min (right side panel). Coomassie staining of the gel shows Histone H3 in all the samples. Recombinant human JAK2 is used as a positive control for the kinase assay.

Next, we addressed whether JAK3 interacts with other nuclear proteins. We hypothesized RNA Polymerase II could be a target as JAK3 was shown to interact with RNA Polymerase II in NK/T-cell lymphoma [45]. Accordingly, we performed co-immunoprecipitation (co-IP) in MyLa2000 and MyLa 2059 and found that JAK3 interacts with POLR2A, the catalytic subunit of RNA Polymerase II (Figure 5a). We verified this interaction by reverse co-IP (Figure 5a) supporting the hypothesis that JAK3 may play a role in nuclear signaling in malignant T cells. As JAK1 and JAK2 modulate epigenetic regulation of gene transcription through tyrosine phosphorylation of the histone protein H3 in leukemic- and B cell lymphoma- cell lines [30,31], we performed in vitro kinase assays to address whether JAK3 was also able to phosphorylate Histone H3. As shown in Figure 5b, recombinant JAK3 induced phosphorylation of recombinant Histone H3, an effect that was inhibited by the JAK inhibitor Tofacitinib citrate. These findings indicated that JAK3 (like JAK1 and JAK2) has the capacity to phosphorylate Histone H3. Further studies are warranted to address whether JAK3 associates with and phosphorylate Histone H3 in vivo and how this might modulate epigenetic regulation and transcription in malignant T cells.

## 3. Discussion

JAK3 belongs to the Janus kinase family of tyrosine kinases, which are believed to play a key role in oncogenesis and disease progression in several cancers including hematological malignancies [46,47,48]. Multiple events and mechanisms including activating mutations in JAKs and STATs have been implicated in the oncogenic role of JAK/STAT signaling [49,50]. Interestingly, mutations in the pseudokinase domain of a particular JAK have been shown to transactivate an opposing different partnering JAK family member [51,52], demonstrating that constitutive activation of JAK3 may also be due to a mutation in a different JAK family member. In addition, mutations of cytokine- and growth factor receptors such as IL7Ra can result in IL-7 receptor hypersensitivity leading to elevated JAK3 activation [53] indicating multilevel control of JAK activation (and deregulation) in cancer.

JAK3 is believed to play a key role in the pathogenesis in CTCL but it is not fully understood what drives aberrant JAK3 in malignant T cells. JAK3 mutations have been described in a small number of CTCL patients indicating a direct role of mutated JAK3 in driving oncogenesis in these patients [54]. Mutations and functional deficiencies in negative regulators of JAK3/STAT3 have also been implicated in the constitutive JAK3/STAT3 signaling in malignant T cells [17,55]. Nuclear expression of JAKs other than JAK3 (such as JAK1, JAK2, and TYK2) have previously been reported in other malignancies and new unexpected–so-called non-canonical–functions (like histone tyrosine phosphorylation) have been assigned to these JAKs [30,36,56]. In contrast, little is known about nuclear expression and function of JAK3 in malignant T cells and their healthy counterpart. Here, we provide first evidence for nuclear expression of JAK3 in malignant T cells. We found JAK3 in the nucleus of malignant T cell lines and primary T cells isolated from peripheral blood of patients with SS, indicating that nuclear JAK3 expression is a common feature of malignant T cells. Whereas nuclear JAK3 expression has not previously been reported in malignant T cells, others have found nuclear JAK3 expression in CD4^+^ T cells from HIV infected patients [32] and in non-hematopoietic cells following exposure of high concentration of IL-2 [57]. These findings suggest that nuclear expression of JAK3 is not an intrinsic feature of malignant T cells, but rather a common phenomenon related to cellular activation. This conclusion is in agreement with other findings indicating a non-canonical, nuclear function of JAKs in epigenetic regulation of transcription [30,31].

As JAKs are not small molecules, they would be expected to have an NLS in order to translocate to the nucleus. Indeed, JAK1 is known to have an NLS sequence required for transport to the nucleus [36]. Using the NLS mapper [37], we were unable to identify NLS in JAK3 suggesting that other mechanisms are involved in nuclear translocation of JAK3. We hypothesized that nuclear translocation of JAK3 might be coupled with translocation of other signaling molecules, e.g., STAT3, which appear to be constantly transported into and out of the nucleus in malignant T cells [35] and accommodate nuclear translocation of other signaling molecules such as NF-kB [58]. In support, STAT3 knockdown by siRNA partly inhibited nuclear expression of JAK3 suggesting that nuclear translocation of JAK3 may partly be coupled with STAT3. Interestingly, nuclear JAK3 localization seemed to be independent of JAK kinase activity and tyrosine phosphorylation of STAT3 as inhibition of kinase activity and STAT3 phosphorylation by Tofacitinib citrate had no effect. This is consistent with the finding that JAK1 activation is not required for its nuclear import [36]. Although it may seem counterintuitive, tyrosine phosphorylation of STAT3 is not essential for its translocation and co-transportation of NF-kB to the nucleus [59]. The present findings suggest that a similar mechanism may be involved in STAT3-mediated translocation of JAK3.

The inhibitor of nuclear export protein CRM1, Leptomycin B, is known to regulate STAT3 expression in the nucleus in certain non-hematopoietic cells [60]. Since STAT3 knockdown had a modest effect on nuclear localization of JAK3 in malignant cells, and because we predicted a potential NES sequence in JAK3, we addressed whether Leptomycin B regulated JAK3 nuclear expression like STAT3. Indeed, Leptomycin B enhanced JAK3 nuclear expression—most profoundly in MyLa2000 cells and weakly in MyLa 2059 cells—supporting the idea that nuclear localization of JAK3 was, at least partly, regulated via CRM1. Of notice, nuclear SOCS3 expression was also increased upon CRM1 inhibition, which was in keeping with our identification of a putative NES sequence in SOCS3. Thus, it appears that all members of the JAK3/STAT3/SOCS3 signaling pathway are regulated by CRM1 implying that JAK3 and SOCS3 may also play a role in nuclear signaling in malignant T cells, which has not been described before. Interestingly, SOCS1 was recently shown to regulate nuclear activity of NF-kB [61] suggesting that SOCS proteins are indeed regulators of nuclear signaling. It is well known that malignant T cells display an ectopic expression of SOCS3, which provides resistance to inhibition by interferon-α [62]. It is also clear that SOCS3 expression is driven by aberrant JAK3/STAT3 activation in malignant T cells [62], whereas a nuclear expression and function of SOCS3 has never been reported in CTCL. Our finding that nuclear JAK3 expression was unaffected by PP2A blockage, which triggered enhanced export of STAT3 as previously reported [35] and confirmed here, suggests that nuclear export of JAK3 in malignant T cells is not regulated in the same manner as STAT3 in relation to PP2A despite the shared regulation by CRM1. CRM1 specific inhibitors are being used in clinical trials as a target for number of cancer therapies including non-Hodgkin lymphomas ([63,64] and reviewed in [65,66]). Of interest, a CRM1 inhibitor showed anti-cancer activity in a JAK3 mutant mouse model for T-ALL [67,68] suggesting the highly interesting possibility that shifting the dynamics of nuclear import/export and the balance between nuclear and cytoplasmic JAK3 might also be a novel potential target for therapy in CTCL. Accordingly, CRM1 inhibitors could be of interest to test as novel remedies for inhibition of malignant T cells in CTCL as it regulates the activity of JAK3/STAT3/SOCS3, a key signaling pathway in malignant transformation in CTCL [17].

As JAK3 is expressed in the nucleus, we investigated the nuclear function of JAK3 in CTCL. Studies have shown that both JAK1 and JAK2 phosphorylates Histone H3 on Tyr 41 [30,31]. We performed in vitro kinase assay and found that recombinant JAK3 phosphorylates recombinant Histone H3 and this phosphorylation was blocked in the presence of JAK inhibitor Tofacitinib citrate. Due to lack of phospho-tyrosine specific antibody against Histone H3, we could not verify the in vivo phosphorylation. As well, we performed co-immunoprecipitation assays and showed that JAK3 interacts with POLR2A, the biggest and catalytic active subunit of RNA Polymerase II. This is consistent with a previous finding by J Yan et al. that JAK3 interacts with RNA Polymerase II in NK/T-cell lymphoma [45]. They proposed that the interaction between JAK3 and RNA Polymerase II was a result of JAK3 interaction with the Histone methyl transferase EZH2, however, we could not find JAK3 interaction with EZH2 in CTCL (data not shown). At present, it remains to be investigated whether nuclear expression of JAK3 involves interaction with POLR2A in healthy, non-malignant T cells or whether this interaction is a unique, cancer-associated feature of malignant T cells.

In conclusion, we provide first evidence that JAK3 is expressed in the nucleus of malignant T cells and interacts with a nuclear protein RNA Polymerase II which supports a non-canonical role of JAK3 in CTCL.

## 4. Materials and Methods 

### 4.1. Cell Lines, Primary Cells and Cell Culture

The cell lines used in the study were previously described [69,70,71,72]. Myla 2059 and Myla 2000 were cultured in RPMI-1640 medium (Sigma, St. Louis, MO, USA #R2405) supplemented with 10% fetal bovine serum (FBS) (Biological Industries, Cromwell, CT, USA #04-007-1A) and 1% Penicillin/Streptomycin (Sigma, #P7539). HH cells were cultured in RPMI-1640 medium supplemented with 20% FBS and 1% Penicillin/Streptomycin. SeAx, was cultured in RPMI-1640 medium supplemented with 10% human serum (HS) (Copenhagen University Hospital Blood Bank), 1% Penicillin/Streptomycin and 1000 U/mL human IL-2 (Novartis, Basel, Switzerland #004184). Cells were maintained in an incubator at 37 °C with 5% CO_2_ and were replenished with fresh medium every two days. Isolation of PBMC from SS patients was done by Ficoll-based density-gradient centrifugation. CD4^+^ T cells were isolated from PBMC using EasySep™ Human CD4^+^ T Cell Isolation Kit (StemCell, Vancouver, BC, Canada #17952) by following the manufacturer’s protocol. Necessary approvals were obtained from the Committee on Health Research ethics (H-16025331) prior to using SS patients’ samples and the work was performed in accordance with the Declaration of Helsinki.

### 4.2. Cell Fractionation and Western Blotting

Cytoplasmic and nuclear lysates were extracted using NE-PER™ Nuclear and Cytoplasmic Extraction Reagents (Thermo Fisher, Waltham, MA, USA #78835) as per manufacturer’s protocol with some modifications. In order to reduce the contamination of the nuclear extract, an additional washing of the nuclear pellet with ice cold Phosphate buffered saline (PBS) was performed after isolating the cytoplasmic extract. Preparation of whole cell lysates, SDS-PAGE and western blotting were described elsewhere [34]. Equal amounts of protein were loaded unless mentioned otherwise.

### 4.3. Transient Transfection

For transient transfections, 2 million cells were re-suspended in a transfection reagent (Mirus Bio, Madison, WI, USA #MIR50111) with 0.2 μM siRNA against either *JAK3* (Dharmacon, Lafayette, CO, USA #L-003147-00-0005) or *STAT3* (Dharmacon, #L-003544-00-0005) or non-targeting control (Dharmacon, #D-001810-01-20) and were transfected using the program A-30 on the Amaxa Nucleofector (Lonza, Basel, Switzerland). Cells were cultured for 48 h after transfection and used for experiments.

### 4.4. Immunofluorescence

Cells were stained on 18 mm diameter coverslips (1.5 thickness) for immunofluorescence. Briefly, 1:1000 dilution of poly-L-lysine (1 mg/mL) solution (Sigma, #P2636) was coated on the coverslips for 30 min and were washed once with PBS. 3 million cells were re-suspended in PBS and were added and dried on top of the coated coverslips for 20 min. Cells were fixed with 2% Paraformaldehyde solution for 15 min, washed gently (three times with PBS for five minutes each) and then permeabilised with ice-cold 100% Methanol (Sigma, #34860) at −20 °C for 12 min. Cells were washed and blocked with 5% Bovine Serum Albumin (Sigma, #A2153) for an hour at room temperature. Primary antibody (anti-JAK3) was added and incubated for an hour at room temperature. Cells were washed three times with PBS and then secondary antibody (goat anti-rabbit Alexa Fluor 488 (Thermo Fisher, #A-11034)) was added at a dilution of 1:700 and incubated for an hour at room temperature. Cells were washed again and then incubated with DAPI (Sigma, #D9542) (1:500 dilution) for 10 min. Coverslips containing cells were mounted on a glass slide (1.5 thickness) using a mounting medium (Thermo Fisher, #S36967), stored at 4 °C until imaging. Cells were then imaged using 63x Plan-Apochromat oil 1.4 numerical aperture (NA) objective of Zeiss LSM710 confocal microscope (Carl Zeiss AG, Oberkochen, Germany).

After immunofluorescence, approximately, 50 randomly chosen cells per treatment per experiment were analysed with Zen Black software (Carl Zeiss AG). The cytoplasm and the nucleus were segmented using Zen Intellesis, an automated intelligent image segmentation feature. Mean fluorescence intensity of JAK3 in the nucleus was then measured and unpaired t-tests were performed to determine statistical significance using Graphpad Prism software (GraphPad, San Diego, CA, USA).

### 4.5. Tofacitinib Citrate Treatment

For JAK inhibition, cells were cultured for 24 h in a 12 well plate treated with 50 μM Tofacitinib citrate (CP-690550-10) (Sigma, #PZ0017) for both MyLa 2059 and MyLa 2000. DMSO (Sigma, #D8418) was used as a control to treat cells without Tofacitinib citrate.

### 4.6. Calyculin A Treatment

MyLa cells were cultured for 24 h and treated with 80 nM Calyculin A (Enzo Life Sciences, Farmingdale, NY, USA #BML-EI192-0025) or DMSO (Control) for 1 h and then, cytoplasmic and nuclear extraction was performed.

### 4.7. Leptomycin B Treatment

To inhibit nuclear export protein CRM1, Leptomycin B solution (Sigma, #L2913) was used. MyLa cells were cultured for 24 h in a 12 well plate and then cells were treated with or without Leptomycin B at concentration ranging between 5 to 20 ng/mL for 3 h. After 3 h, cytoplasmic and nuclear fractions were extracted.

### 4.8. Co-Immunoprecipitation

For co-immunoprecipitation (co-IP), Pierce™ Protein A Magnetic Beads (Thermo Fisher, #88845) were used and the experiments were performed according to the manufacturer’s protocol with slight modifications. Briefly, pre-cleared whole cell lysates were incubated overnight with primary antibody at 4 °C with constant mixing. Then, pre-washed protein A beads were added to the antigen-antibody complex and mixed at room temperature for an hour. The antigen-antibody complex bound beads were separated from the mix using the DynaMag™-2 Magnet (Thermo Fisher, #12321D) and were washed three times with Tris-buffered Saline (TBS) containing 0.01% Tween-20 (Sigma, #822184). The beads were washed once with sterile water. Then bound proteins were eluted by mixing beads with 2× Laemmli SDS sample buffer and were denaturized at 100 °C for 10 min in a heat block. SDS-PAGE and western blotting experiments were then performed. 

### 4.9. Radioactive In Vitro Kinase Assay

For in vitro kinase assay, 1 ng of human recombinant JAK3 active protein (Sigma, #14-629), 1 ng of human recombinant JAK2 active protein (Sigma, #14-640) and 2 μg of human recombinant Histone H3 (Sigma, #14-494) were used. Briefly, the kinase (JAK2 or JAK3) and the substrate (Histone H3) were re-suspended in a kinase buffer (20mM TRIS-HCl pH 7.5, 5 mM MgCl_2_, 5mM MnCl_2_, 1 μM cold ATP) containing 5 μCi γ-^32^P labelled ATP (Perkin Elmer, Waltham, MA, USA #NEG002A100UC) and were incubated at 37 °C for 30 min. The reaction was stopped by adding Laemmli SDS sample buffer and the samples were kept on ice immediately. For inhibition of JAK3, 100 nM Tofacitinib citrate was pre-incubated with the kinase for 30 min at room temperature. The kinase and the substrate were separated by running samples on 4–20% SDS-PAGE gel (BioRad, Hercules, CA, USA) followed by staining gel with Coomassie Brilliant blue (BioRad, #1610786). The gel was de-stained, dried, and kept in contact with storage phosphor image screen (GE Lifesciences, Marlborough, MA, USA) and the screen was visualized in a Typhoon Phosphorimager (GE Lifesciences).

### 4.10. Antibodies

JAK3-1:1000 (Cell Signaling Technology, Danvers, MA, USA #8827), JAK3-1:1000 for western blotting and 1:500 for immunofluorescence (Abcam, Cambridge, UK #ab45141), JAK3-1:100 for co-IP (Santa Cruz Biotechnology, Dallas, TX, USA #sc-513), JAK3-1:1000 used for western blotting followed by co-IP (Novus Biologicals, Centennial, CO, USA #NBP2-37737), p-STAT3 (Y705)-1:1000 (Cell Signaling Technology, #9145), GAPDH-1:10,000 (Santa Cruz Biotechnology, #sc-32233), Lamin A/C-1:1000 (Cell Signaling Technology, #2032), POLR2A-1:1000 (BioLegend, San Diego, CA, USA #664906), anti-mouse HRP-1:2000 (Agilent Dako, Glostrup, Denmark #P044701-2), anti-rabbit HRP-1:1000 (Agilent Dako, #P044801-2), Isotype controls-rabbit IgG (Cell signaling Technology, #3900) and mouse IgG2A (R&D Systems, Minneapolis, MN, USA #MAB0031).

## 5. Conclusions

In this study, we provide first evidence that JAK3 is expressed in the nucleus of primary malignant T cells and T cell lines from patients with in CTCL. Nuclear translocation of JAK3 was independent of its kinase activity and the nuclear import and export of JAK3 was regulated differently from other members of the JAK/STAT/SOCS signaling cascade. Importantly, JAK3 interacts with the nuclear protein POLR2A, the catalytic subunit of RNA Polymerase II and kinase assays showed tyrosine phosphorylation of recombinant human Histone H3 by JAK3 in vitro. These results suggest that JAK3 may have a novel, non-canonical role in the nucleus in malignant cells.

## Data Availability

The data presented in this study are available in the manuscript and in the Supplementary Materials.

## Supplementary Materials

The following are available online at https://www.mdpi.com/2072-6694/13/2/280/s1, Figure S1: JAK3 expression in MyLa 2000. Figure S2: JAK3 expression in healthy individuals. Figure S3: Immunoblots corresponding to Figure 1 and Figure 2, Figure S4: Immunoblots corresponding to Figure 3 and Figure 4. Figure S5: Immunoblots corresponding to Figure 5, Figures S1 and S2. Table S1: JAK3 regulated genes in MyLa 2059. Table S2: Relative expression of JAK3, Table S3: Relative expression of STAT3, Table S4: Relative expression of SOCS3. Video S1: 3-dimensional view from top to bottom of a single cell after JAK3 immunofluorescence staining in MyLa 2059 corresponding to Figure 1c. Video S2–S4: 3-dimensional view from top to bottom of a single cell after JAK3 immunofluorescence staining in SS patient 1 (Video S2), SS patient 2 (Video S3), and SS patient 3 (Video S4) corresponding to Figure 2c. The videos were made using Amira (Thermo Fisher).
