# Supplementary material for: JAK3 Is Expressed in the Nucleus of Malignant T Cells in Cutaneous T Cell Lymphoma (CTCL)
Source: Cancers (Basel). 2021 Jan 14;13(2):280. doi: 10.3390/cancers13020280 (PMC7828698; doi:10.3390/cancers13020280)
Supplement: Supplementary file 1 [file cancers-13-00280-s001.zip › cancers-983688-supplmentary-final/cancers-983688-supplementary-final.pdf]

cancers

MDPI

Supplementary Materials: JAK3 Is Expressed in the Nucleus of Malignant T Cells in Cutaneous T Cell Lymphoma (CTCL)

Chella Krishna Vadivel, Maria Gluud, Sara Torres-Rusillo, Lasse Boding, Andreas Willerslev-Olsen, Terkild B. Buus, Tea Kirkegaard Nielsen, Jenny L. Persson, Charlotte M. Bonefeld, Carsten Geisler, Thorbjorn Krejsgaard, Anja T. Fuglsang, Niels Odum and Anders Woetmann

Table S1. JAK3 regulated genes in MyLa 2059.

JAK3 regulated genes	Fold Change	Role in CTCL	References
IL-9	53.3	Increases tumor cell proliferation	[1]
miR-155	7.0	Promotes tumor proliferation, repression of tumor suppressors and disease progression	[2]
SOCS3	6.2	Resistance to IFN alpha	[3]
LTA	5.4	Promotes IL-6 and involved in malignant angiogenesis	[4]
IL-17F	4.0	Promotes angiogenesis and associated with disease progression	[5]
miR-21	2.3	Shaping of TME	[6]
IL-5	2.2	Promotes a TH2 biased TME and eosinophilia	[7]
STAT3	2.0	Proto-oncogene that promotes survival of malignant T cells and expression of inflammatory cytokine and mRNAs	[8]
CD80	1.5	Suppresses anti-cancer immunity	[9]
STAT4	−1.5	Loss of STAT4 is associated with deficient TH1 and a shift towards a TH2 profile in the TME	[10]

Table S2. Relative expression of JAK3. Densitometry values of JAK3 relative to GAPDH or Lamin A/C calculated using Image J for western blots shown in figures.

Experiment	Condition	JAK3	Statistics
Figure 1a MyLa 2000	C	1	
	N	0.67	
Figure 1a MyLa 2059	C	1	
	N	0.6	
Figure 1a SeAx	C	1	
	N	0.43	
Figure 1a HH	C	1	
	N	0.3	
Figure 1b JAK3 transient knockdown MyLa 2059	NT siRNA (−) / JAK3 siRNA (−) C (Lane 1)	1	Ordinary one-way ANOVA analysis, $p < 0.05$
	NT siRNA (+) / JAK3 siRNA (−) C (Lane 2)	0.9	
	NT siRNA (−) / JAK3 siRNA (+) C (Lane 3)	0.01	
	NT siRNA (−) / JAK3 siRNA (−) N (Lane 4)	0.8	
	NT siRNA (+) / JAK3 siRNA (−) N (Lane 5)	1	
	NT siRNA (−) / JAK3 siRNA (+) C (Lane 6)	0	

Figure 2a PBMC from SS patients SS1	C	1	
	N	0.96	
Figure 2a PBMC from SS patients SS2	C	1	
	N	0.5	
Figure 2a PBMC from SS patients SS3	C	1	
	N	0.3	
Figure 2b CD4+ T cells from SS patients SS1	C	0.9	
	N	1	
Figure 2b CD4+ T cells from SS patients SS2	C	1	
	N	0.6	
Figure 3a Tofacitinib Treatment MyLa 2000	DMSO	0.97	Unpaired t-test, p value non-significant
	JAKi	1	
Figure 3a Tofacitinib Treatment MyLa 2059	DMSO	1	Unpaired t-test, p value non-significant
	JAKi	0.84	
Figure 3b STAT3 transient knockdown MyLa 2000	NT siRNA (+) / STAT3 siRNA (−) (Lane 1)	1	Unpaired t-test, $p < 0.05$
	NT siRNA (−) / STAT3 siRNA (+) (Lane 2)	0.56	
Figure 3b STAT3 transient knockdown MyLa 2059	NT siRNA N (Lane 2)	1	Unpaired t-test, $p < 0.05$
	STAT3 siRNA N (Lane 4)	0.55	
Figure 4a Leptomycin B treatment MyLa 2000	0	0.53	Ordinary one-way ANOVA analysis, $p < 0.05$
	5	0.84	
	10	0.77	
	15	1	
	20	0.82	
Figure 4a Leptomycin B treatment MyLa 2059	0	0.52	Ordinary one-way ANOVA analysis, $p < 0.05$
	5	0.74	
	10	0.75	
	15	1	
	20	0.82	
Figure 4b Calyculin A treatment MyLa 2000	Control N (Lane 2)	0.96	Unpaired t-test, p value non-significant
	Calyculin N (Lane 4)	1	
Figure 4b Calyculin A treatment MyLa 2059	Control N (Lane 2)	0.93	Unpaired t-test, p value non-significant
	Calyculin N (Lane 4)	1	
Figure S1 JAK3 transient knockdown MyLa 2000	NT siRNA (+) / JAK3 siRNA (−) C (Lane 1)	1	Unpaired t-test, $p < 0.05$
	NT siRNA (−) / JAK3 siRNA (+) C (Lane 2)	0.01	
	NT siRNA (+) / JAK3 siRNA (−) N (Lane 3)	1	

	NT siRNA (−) / JAK3 siRNA (+) N (Lane 4)	0	
Figure S2 Healthy individuals PBMC #2	C	1	
	N	0.4	
Figure S2 Healthy individuals PBMC #3	C	1	
	N	0.13	
Figure S2 Healthy individuals $CD4^+$ T cells #4	C	1	
	N	0.5	
Figure S2 Healthy individuals $CD4^+$ T cells #5	C	1	
	N	0.3	

Table S3. Relative expression of STAT3. Densitometry values of STAT3 relative to Lamin A/C calculated using Image J for western blots shown in figures in the main text.

Experiment	Condition	STAT3	Statistics
Figure 3b STAT3 transient knockdown MyLa 2000	NT siRNA (+) / STAT3 siRNA (−) (Lane 1)	1	Unpaired t-test, $p < 0.05$
	NT siRNA (−) / STAT3 siRNA (+) (Lane 2)	0.4	
Figure 3b STAT3 transient knockdown MyLa 2059	NT siRNA N (Lane 2)	1	Unpaired t-test, $p < 0.05$
	STAT3 siRNA N (Lane 4)	0.04	
Figure 4b Calyculin A treatment MyLa 2000	Control N (Lane 2)	1	Unpaired t-test, $p < 0.05$
	Calyculin N (Lane 4)	0.4	
Figure 4b Calyculin A treatment MyLa 2059	Control N (Lane 2)	1	Unpaired t-test, $p < 0.05$
	Calyculin N (Lane 4)	0.2	

Table S4. Relative expression of SOCS3. Densitometry values of SOCS3 relative to Lamin A/C calculated using Image J for western blots shown in figures in the main text.

Experiment	Condition	SOCS3	Statistics
Figure 4a Leptomycin B treatment MyLa 2000	0	0.2	Ordinary one-way ANOVA analysis, $p < 0.05$
	5	0.81	
	10	0.56	
	15	1	
	20	0.92	
Figure 4a Leptomycin B treatment MyLa 2059	0	0.17	Ordinary one-way ANOVA analysis, $p < 0.05$
	5	1	
	10	0.89	
	15	0.81	
	20	0.75	

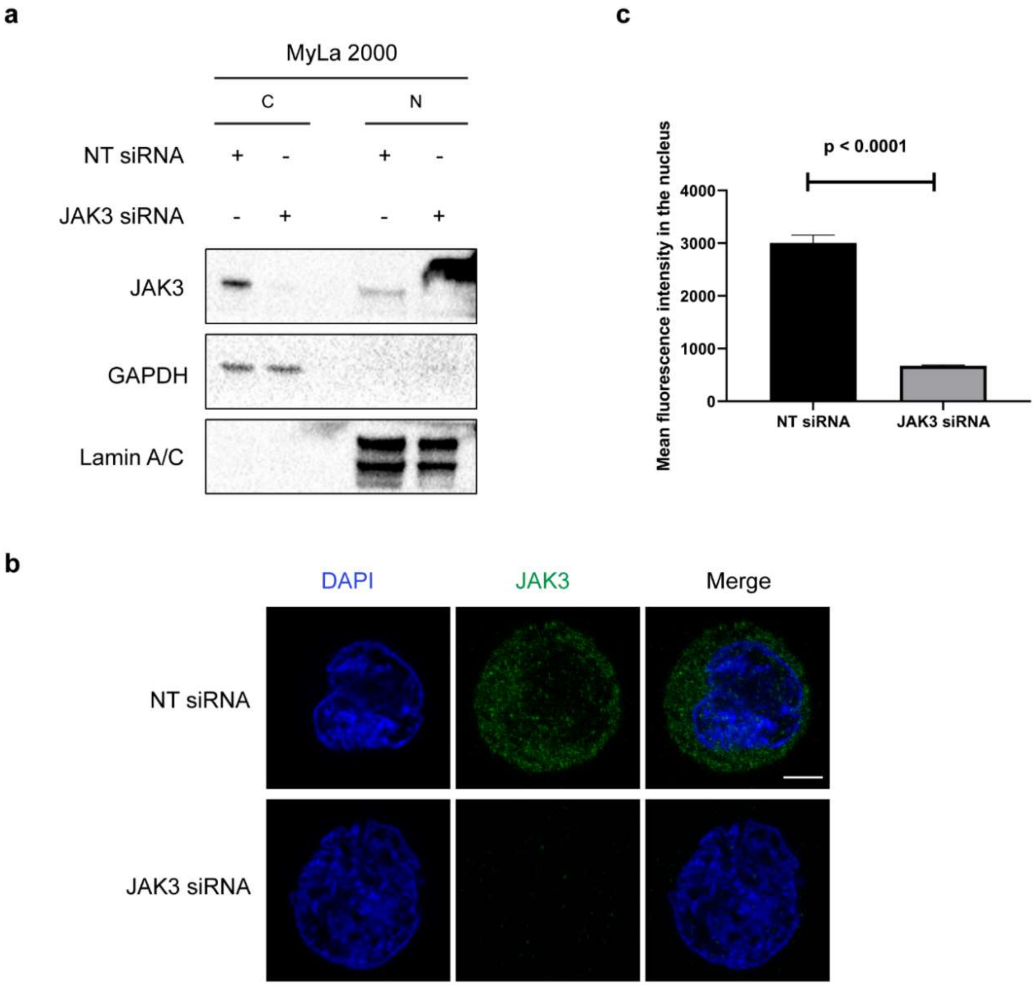

Figure S1. JAK3 expression in MyLa 2000. (**a**) Immunoblots show low or no expression of JAK3 in the cytoplasmic (C) and nuclear (N) extracts in MyLa 2000 after 48 hours of siRNA mediated knockdown of JAK3. (**b**) Immunofluorescence (IF) of MyLa 2000 confirming JAK3 expression in the nucleus. Scale bar = 5 µm. NT siRNA is the non-targeted control siRNA and JAK3 siRNA is the siRNA targeted against *JAK3*. (**c**) Mean fluorescence intensity of JAK3 calculated in the nucleus of MyLa 2000 after IF. Error bar represents standard error of mean.

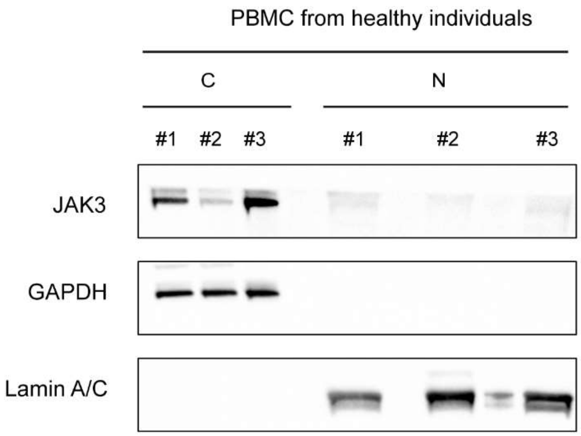

Figure S2. JAK3 expression in PBMC from healthy individuals. Immunoblots show JAK3 expression in cytoplasmic (C) and nuclear (N) extracts of PBMC isolated from three healthy individuals.

Figure 1

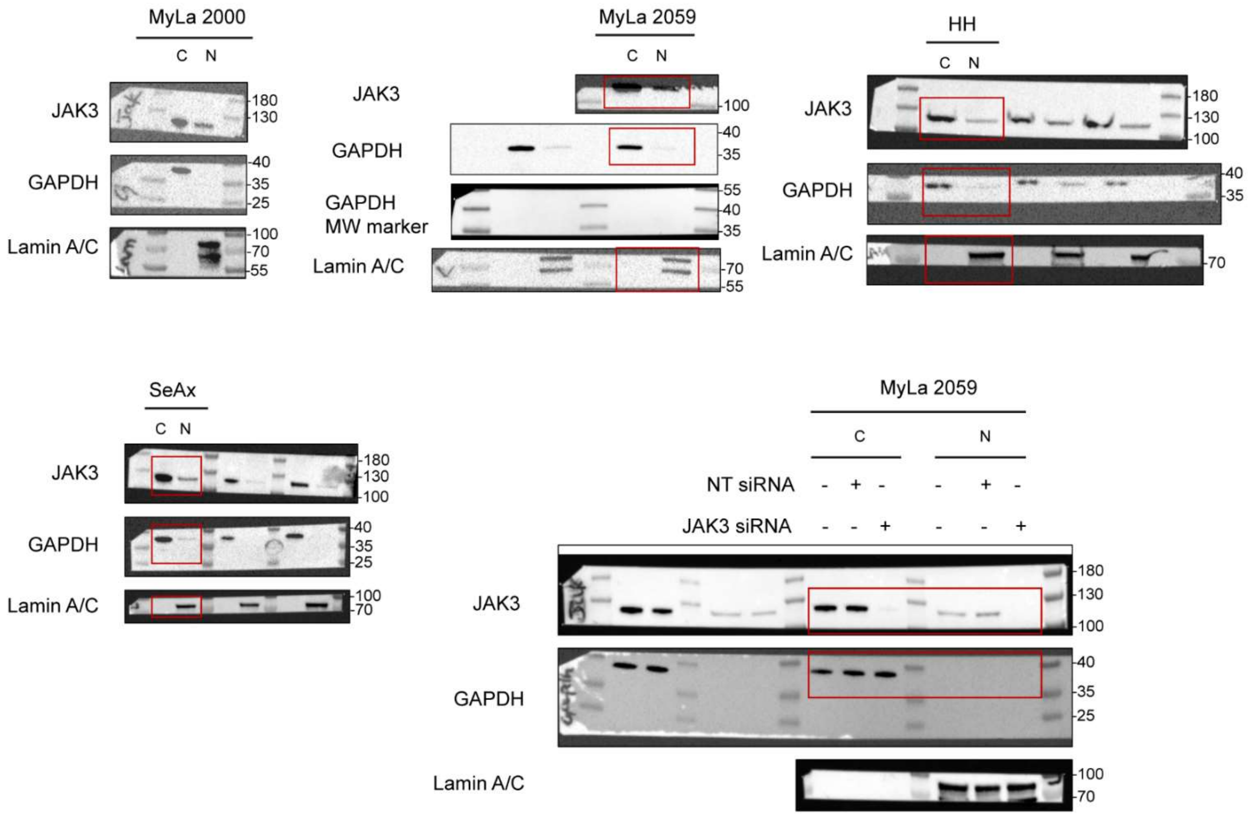

Figure 2

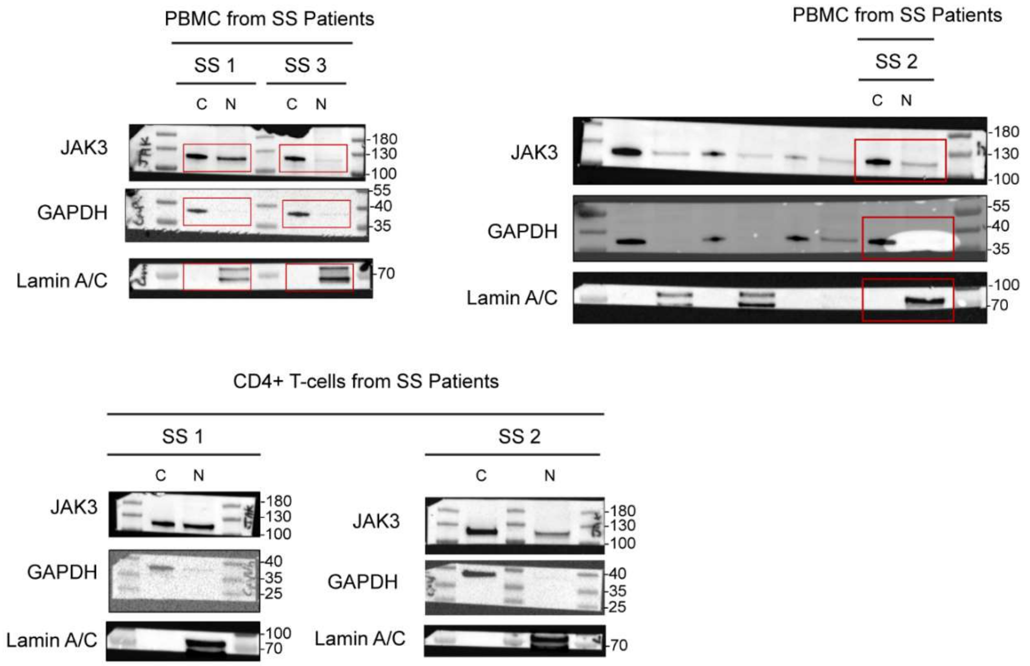

Figure S3. Immunoblots corresponding to Figures 1 and 2. Red boxes indicate the bands used in the figures. The numbers on the right side of each blot represents the molecular weight (MW) in KDa.

Figure 3

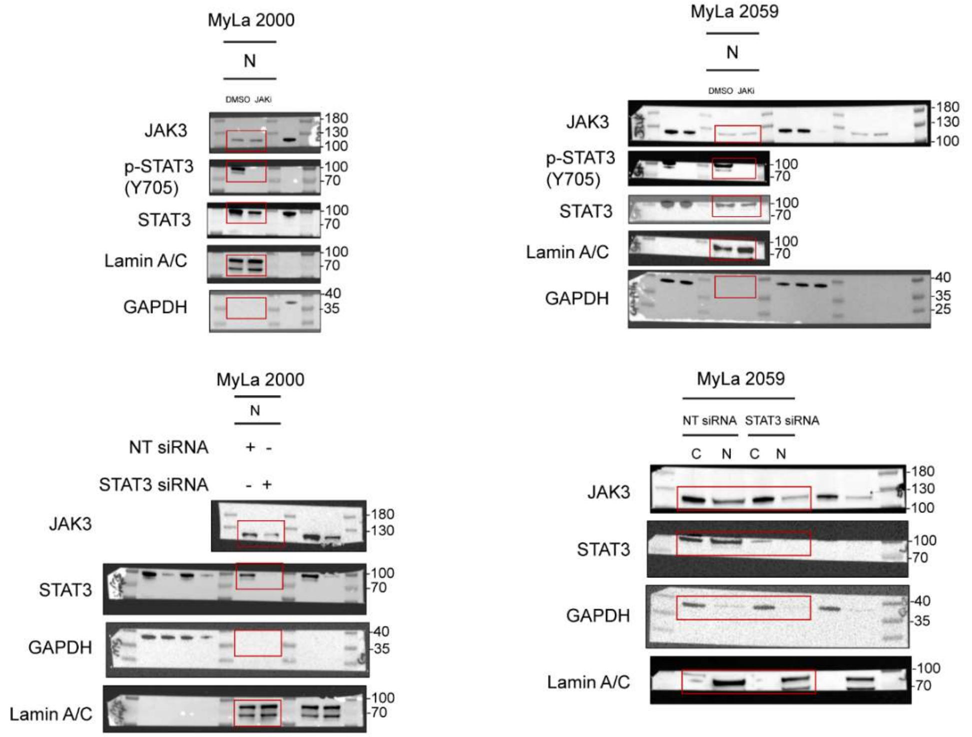

Figure 4

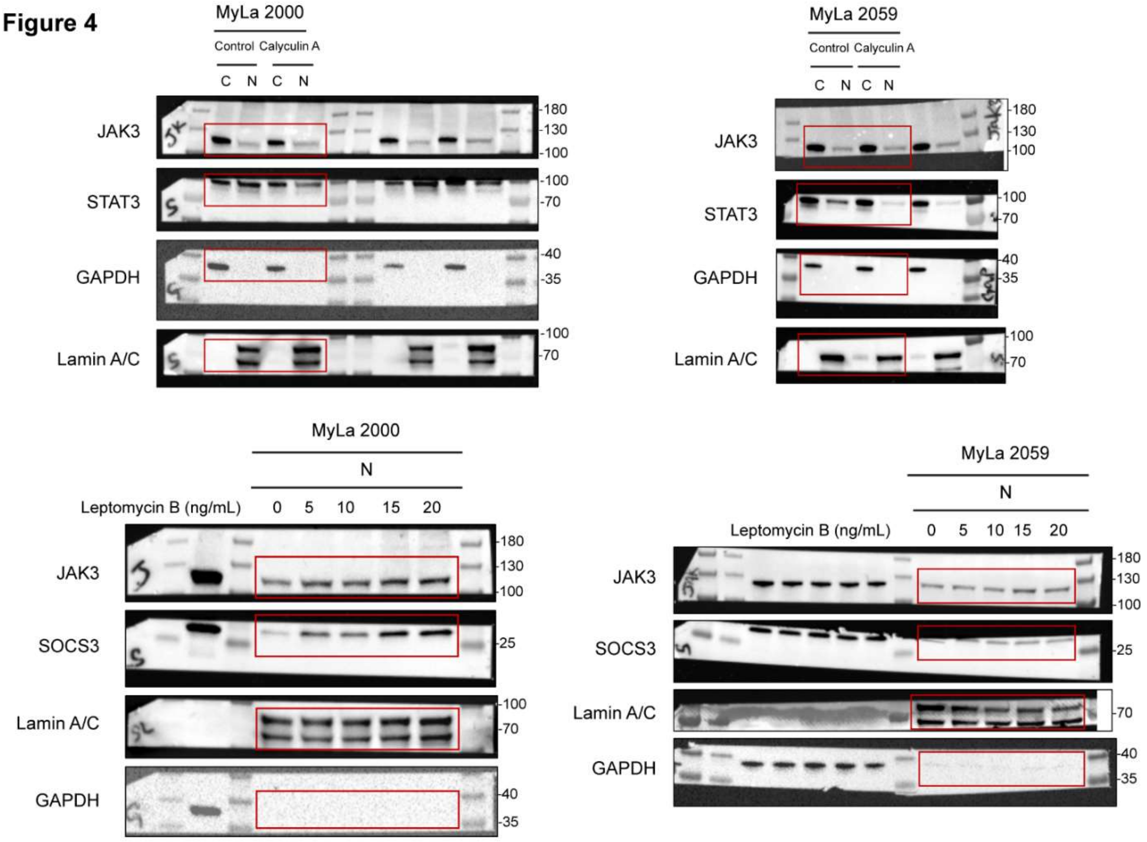

Figure S4. Immunoblots corresponding to Figures 3 and 4. Red boxes indicate the bands used in the figures in the main text. The numbers on the right side of each blot represents the molecular weight in KDa.

Figure 5

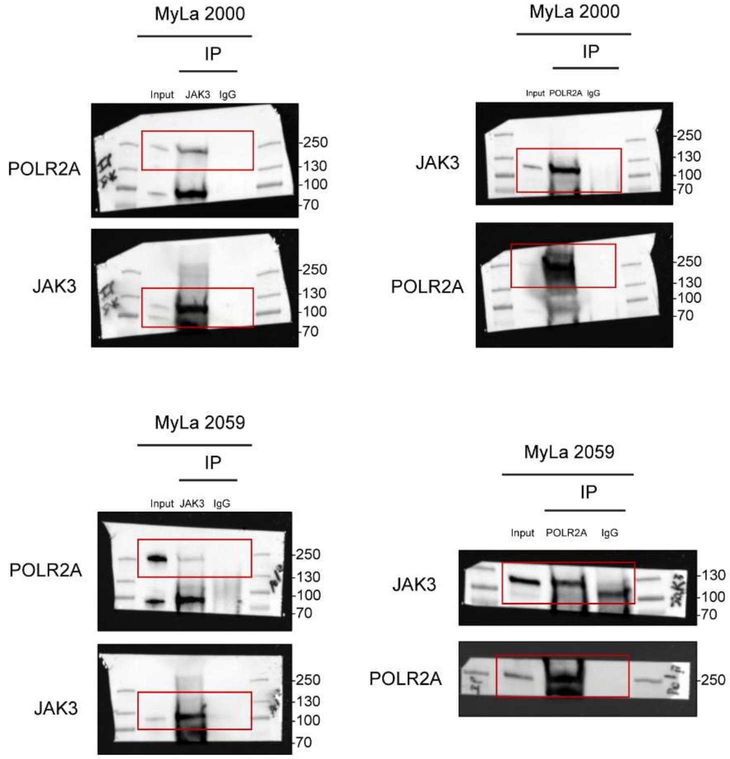

Figure S1

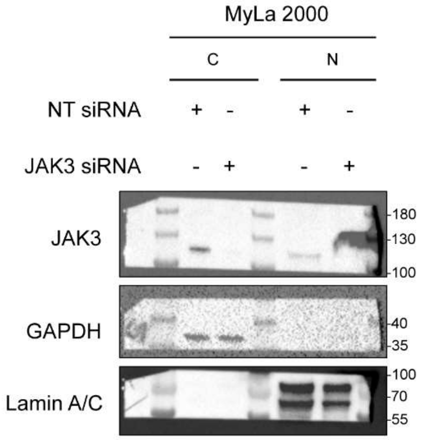

Figure S2

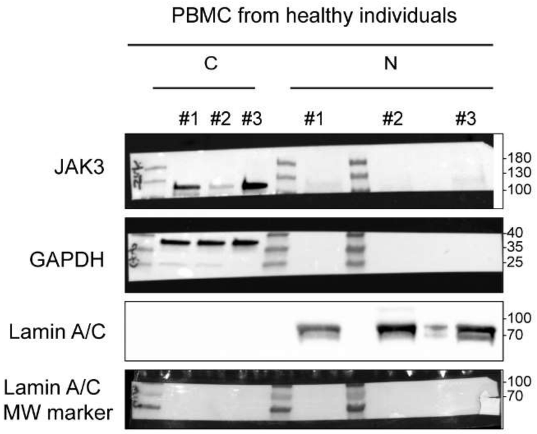

Figure S5. Immunoblots corresponding to Figures 5, S1 and S2. Red boxes indicate the bands used in the figures in the main text. The numbers on the right side of each blot represents the molecular weight (MW) in KDa.

References

1. Vieyra-Garcia, P.A.; Wei, T.; Naym, D.G.; Fredholm, S.; Fink-Puches, R.; Cerroni, L.; Odum, N.; O'Malley, J.T.; Gniadecki, R.; Wolf, P. STAT3/5-Dependent IL9 Overexpression Contributes to Neoplastic Cell Survival in Mycosis Fungoides. *Clin. Cancer Res.* **2016**, *22*, 3328–3339, doi:10.1158/1078-0432.CCR-15-1784.
2. Moyal, L.; Barzilai, A.; Gorovitz, B.; Hirshberg, A.; Amariglio, N.; Jacob-Hirsch, J.; Maron, L.; Feinmesser, M.; Hodak, E. miR-155 is involved in tumor progression of mycosis fungoides. *Exp. Dermatol.* **2013**, *22*, 431–433, doi:10.1111/exd.12161.
3. Brender, C.; Lovato, P.; Sommer, V.H.; Woetmann, A.; Mathiesen, A.M.; Geisler, C.; Wasik, M.; Ødum, N. Constitutive SOCS-3 expression protects T-cell lymphoma against growth inhibition by IFNα. *Leukemia* **2005**, *19*, 209–213, doi:10.1038/sj.leu.2403610.

4. Lauenborg, B.; Christensen, L.; Ralfkiaer, U.; Kopp, K.L.; Jønson, L.; Dabelsteen, S.; Bonefeld, C.M.; Geisler, C.; Gjerdrum, L.M.; Zhang, Q.; et al. Malignant T cells express lymphotoxin α and drive endothelial activation in cutaneous T cell lymphoma. *Oncotarget* **2015**, *6*, 15235–15249, doi:10.18632/oncotarget.3837.
5. Ehrentraut, S.; Schneider, B.; Nagel, S.; Pommerenke, C.; Quentmeier, H.; Geffers, R.; Feist, M.; Kaufmann, M.; Meyer, C.; Kadin, M.E.; et al. Th17 cytokine differentiation and loss of plasticity after SOCS1 inactivation in a cutaneous T-cell lymphoma. *Oncotarget* **2016**, *7*, 34201–34216, doi:10.18632/oncotarget.9077.
6. Lindahl, L.M.; Fredholm, S.; Joseph, C.; Nielsen, B.S.; Jønson, L.; Willerslev-Olsen, A.; Gluud, M.; Blümel, E.; Petersen, D.L.; Sibbesen, N.; et al. STAT5 induces miR-21 expression in cutaneous T cell lymphoma. *Oncotarget* **2016**, *7*, 45730–45744, doi:10.18632/oncotarget.10160.
7. Nielsen, M.; Nissen, M.H.; Gerwien, J.; Zocca, M.B.; Rasmussen, H.M.; Nakajima, K.; Röpke, C.; Geisler, C.; Kaltoft, K.; Ødum, N. Spontaneous interleukin-5 production in cutaneous T-cell lymphoma lines is mediated by constitutively activated Stat3. *Blood* **2002**, *99*, 973–977, doi:10.1182/blood.v99.3.973.
8. van Kester, M.S.; Out-Luiting, J.J.; von dem Borne, P.A.; Willemze, R.; Tensen, C.P.; Vermeer, M.H. Cucurbitacin I inhibits Stat3 and induces apoptosis in Sézary cells. *J. Invest. Dermatol.* **2008**, *128*, 1691–1695, doi:10.1038/sj.jid.5701246.
9. Zhang, Q.; Wang, H.Y.; Wei, F.; Liu, X.; Paterson, J.C.; Roy, D.; Mihova, D.; Woetmann, A.; Ptasznik, A.; Odum, N.; et al. Cutaneous T cell lymphoma expresses immunosuppressive CD80 (B7-1) cell surface protein in a STAT5-dependent manner. *J. Immunol.* **2014**, *192*, 2913–2919, doi:10.4049/jimmunol.1302951.
10. Litvinov, I.V.; Cordeiro, B.; Fredholm, S.; Ødum, N.; Zargham, H.; Huang, Y.; Zhou, Y.; Pehr, K.; Kupper, T.S.; Woetmann, A.; et al. Analysis of STAT4 expression in cutaneous T-cell lymphoma (CTCL) patients and patient-derived cell lines. *Cell Cycle* **2014**, *13*, 2975–2982, doi:10.4161/15384101.2014.947759.
